# Does the Paleo diet affect an athlete’s health and sport performance?

**DOI:** 10.5114/biolsport.2023.123325

**Published:** 2023-02-08

**Authors:** Barbara Frączek, Aleksandra Pięta

**Affiliations:** 1 Department of Sports Medicine and Human Nutrition, Institute of Biomedical Sciences, University School of Physical Education in Kraków, Poland

**Keywords:** Carbohydrate diet, Paleolithic diet, Alternative diet, Exercise capacity, Body composition

## Abstract

The aim of the study was to assess the impact of an eight-week Paleo diet on the health status (body composition, haematology and biochemistry of blood and urine) and the level of physical capacity (aerobic and anaerobic) of professional handball players. Fifteen athletes were assigned to two groups: 9 in the experimental group (PD) and 6 in the control group (CD). Significant decreases in body mass (BM), body mass index (BMI), and fat mass (FM) as well as an increase in the fat-free mass (FFM) (%) in both groups were observed. There were no significant differences between groups in particular series during the experiment in all haematological and biochemical indicators of blood and urine. Only HDL-C was significantly higher in the last series in the PD compared to the CD (1.63 mmol/l vs. 1.23 mmol/l). In the Wingate test, there were only single intragroup changes, consisting of a significant decrease in the Wt, MAP and Pmean in the experimental group. There were no significant differences between the groups in individual series or intragroup differences during the experiment, determined by the VO_2_max, VEmax, VE ∙ VCO_2_^−1^, RER, and the time of the test with a gradually increasing load on a treadmill, except for a significant decrease of maximum tidal volume (TVmax) in the PD. No adverse effect of the Paleo diet on the health status was found. The use of the Paleo diet slightly adversely affects anaerobic capacity and does not affect the level of aerobic capacity.

## INTRODUCTION

Paleolithic nutrition is based on the principles of evolutionary biology with a focus on the low carbohydrate options available to hunter-gatherers. The Paleo diet consists mainly of grass-fed and pasture-raised meats, vegetables, fruits, fungi, roots and nuts, excludes grains, legumes and dairy products, and limits refined sugars, starches, processed foods and oils. The Paleo diet is defined by the avoidance of particular food sources rather than a specific macronutrient distribution [1].

There are many scientific articles that evaluate effects of the Paleo diet (PD) on health status in people with diseases, such as ischemic heart disease [2], blood lipid disorder [3], overweight or obesity [4–5], diabetes [2, 4, 6], and metabolic syndrome [7], and even in healthy, inactive adults [8, 9]. Most, albeit not all, studies suggest that a PD has positive effects on body composition [2–5, 7, 9], insulin sensitivity and/or fasting blood glucose [2, 3, 7, 8], blood lipids [3, 4, 7–9], and blood pressure [4, 7, 8]. Only a few studies have examined the effects of a PD with exercise on cardiorespiratory fitness in healthy or unhealthy adults [5, 6, 9].

On average, the authors estimate the following ratio of macronutrients: 35% energy from fats, 35% from carbohydrates, and 30% from protein (although no specific amount is the goal) [7]. Currently, researchers evaluating the nutritional value of the Paleo diet classify it as a moderate-carbohydrate diet [10]. There are many studies determining the impact of a diet with very low amounts of carbohydrates (e.g., ketogenic diet; KD) on athletic performance, while there are only several research trials using a moderate carbohydrate diet in a group of athletes [11]. So far, no scientific studies have been carried out to evaluate the use of the Paleo diet among athletes, despite this diet being currently a very fashionable nutrition strategy willingly used by athletes. It should be noted that the relatively small (but sufficient) amount of carbohydrates in the Paleo diet is increasingly used by athletes with the aim of using fat during exercise and saving carbohydrate reserves. That is why we became interested in this subject and decided to start research assessing the use of the Paleo diet in the context of sports. Considering the athletes’ willingnes to the use of the Paleo diet, we wanted to assess the impact of an eight-week Paleo diet on the health status (body composition, haematology and biochemistry of blood and urine) and the level of physical capacity (aerobic and anaerobic) of professional handball players.

## MATERIALS AND METHODS

### Characteristics of participants

At the beginning, 22 professional handball players training competitively were invited to take part in the experiment involving the application of an eight-week nutritional intervention. Twenty-two athletes who competed in a local, 2^nd^ league team in Kraków were recruited. The inclusion criteria in the study group were: age 18–25 years, at least 5 years of participation in competitive sports, undertaking regular physical activity at least 5 times a week for more than one hour per training session, and participation in national and/or international competitions. Five participants dropped out of the experiment in the first research series as they failed to maintain the nutritional regime, regardless of the diet they were to use, despite the high taste preference for the diet or a rational and expressed willingness to take up the diet indicated in the questionnaire. Two athletes ultimately failed to meet the inclusion criterion for undertaking physical activity for all subjects. Finally, 15 athletes completed the full-time study. Based on the analysis of taste preferences and after expressing their willingness to undertake a rational or Paleo diet, the competitors were assigned to two groups differing in the type of diet: 9 in the experimental group and 6 in the control group. At baseline, the two groups did not differ in terms of age [experimental group: 22.00 (21.00; 24.00), control group: 24.5 (23.25; 26.50)] or in anthropometric parameters [body height: 1.86 (1.81; 1.95) cm and 1.85 (1.81; 1.89) cm, respectively, and body mass: 91.36 (87.5; 99.50) kg and 88.4 (84.7; 100.65) kg, respectively].

### Dietary intervention

To determine the individual total energy demand (ED), before starting the experiment, the players performed 10-day monitoring of total energy expenditure (on 8 training days and 2 non-training days) using a heart rate monitor (Polar M400/RS400) [12]. Median energy expenditure (EE) in the experimental group was: 4288 (4127; 5000) kcal on training days and 3400 (2900; 3412) kcal on non-training days, and accordingly in the control group: 4096 (3853; 4775) kcal and 3040 (2860; 3646) kcal. There were no statistically significant differences in EE. The ED in both groups was determined considering a sustainable energy balance.

Based on the determined energy demand and the individual needs for nutrients, body mass and food preferences, two nutritional strategies have been developed: the rational diet (control diet; CD), in accordance with the current nutritional recommendations for active people [12, 13], and the Paleo diet (PD) based on the qualitative recommendations of the nutritional model [1]. The PD included grass-fed and pasture-raised meats, vegetables, fruits, fungi, roots and nuts, excluded grains, dairy products and legumes, and limited refined sugars and highly processed foods [1]. The all-day food rations were prepared using the “Aliant. Dietetic Calculator” 4.10.14 (Cambridge Diagnostics, Poland) considering individual assumptions developed by the researcher, considering the isocaloric model of the diet. A different energy supply was used on training/match days (training sessions were held from Monday to Friday, and matches were held on Saturdays) and on a day off training, intended for regeneration. For a rational diet, the following energy share of macronutrients was adopted: 15% protein (no more than 20%, depending on body mass; BM), 25% fat (no more than 30%, depending on BM) and approximately 55% carbohydrates (no more than 60%, depending on BM) [13, 14], and for the Paleo diet, accordingly, 30% protein (not less than 20%, depending on BM), 40% fat (not less than 35%, depending on BM) and 30% of carbohydrates (no more than 35%, depending on BM) [8]. A total of 840 daily food rations were created: 504 for the subjects in the experimental group and 336 in the control group, 56 for each subject, which were then prepared by the catering company. The diets were used by competitors for 56 days (eight weeks). During the experiment, the athletes did not take any supplements influencing the resting and exercise metabolism.

### Experimental design

The research was conducted in accordance with the Declaration of Helsinki and the research methodology was approved by the Bioethical Committee of the Regional Medical Chamber in Kraków (38/KBL/OIL/2017). The participants were informed in detail about the purpose and course of the study and about the possibility to withdraw from participation in the project at any stage without providing a reason. All the subjects read the written information about the research course, especially the nutritional strategy. The participants provided their written consent for voluntary participation in the trial.

**FIG. 1:**
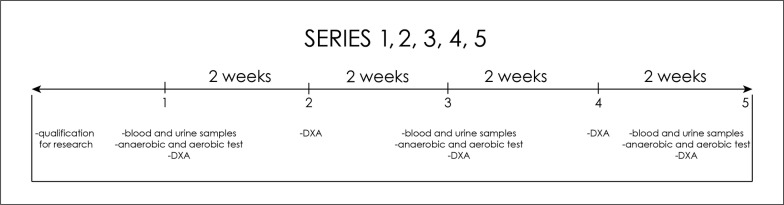
Five measurement series.

Five measurement points (series) were distinguished: 1 – before the start of nutritional intervention or on the first day of the experiment; 2 – after two weeks of nutritional intervention; 3 – after four weeks of nutritional intervention; 4 – after six weeks of nutritional intervention; 5 – after eight weeks of nutritional intervention (Figure 1). Before starting the experiment and after the 2^nd^, 4^th^, 6^th^ and 8^th^ week, the body composition was measured using dual energy X-ray absorptiometry (DXA) using the Lunar iDXA instrument. Fat mass (FM) [% and kg], fat free mass (FFM) [% and kg], lean body mass (body mass without adipose tissue and bone minerals: (LBM) [% and kg], bone mineral content (BMC) [g] and bone mineral density (BMD) [g/cm^2^] were analysed. The content of muscle mass (MM) [% and kg] was calculated using the Kim formula [15].

In order to assess the health effects of the Paleo diet, on the first day of the study, on an empty stomach, and on the first day of the week after weeks 2, 4, 6 and 8, a qualified nurse took blood samples from the veins in the elbow pit to determine haematological and biochemical blood indicators, i.e. peripheral blood counts (haematocrit, haemoglobin concentration, erythrocyte count, platelets, white blood cell count – leukocytes, leukocyte differentiation: lymphocytes, monocytes, basophils, eosinophils, neutrophils), glucose, insulin, total cholesterol, high-density lipoprotein (HDL) cholesterol, low-density lipoprotein (LDL) cholesterol, triglycerides (TG), creatinine (Cr), urea (U), uric acid (UA), sodium (Na) and potassium (K), calcium (Ca), magnesium (Mg), liver enzymes: ALT (alanine aminotransferase), AST (aspartic aminotransferase), alkaline phosphatase and GGTP (gamma glutamyltransferase), cystatin C, total bilirubin, free fatty acids (FFA) and beta-hydroxybutyrate (b-HB) (Figure 1). On the 1^st^ day of the tests and after the 2^nd^, 4^th^, 6^th^ and 8^th^ weeks, the competitors also left samples of early morning urine to assess their health and acidification levels. Total urine tests were performed, i.e., specific gravity, pH, leukocytes, nitrites, protein, glucose, ketones, urobilinogen, bilirubin, blood (erythrocytes/haemoglobin), colour, transparency as well as sodium, potassium, and creatinine levels.

**FIG. 2:**
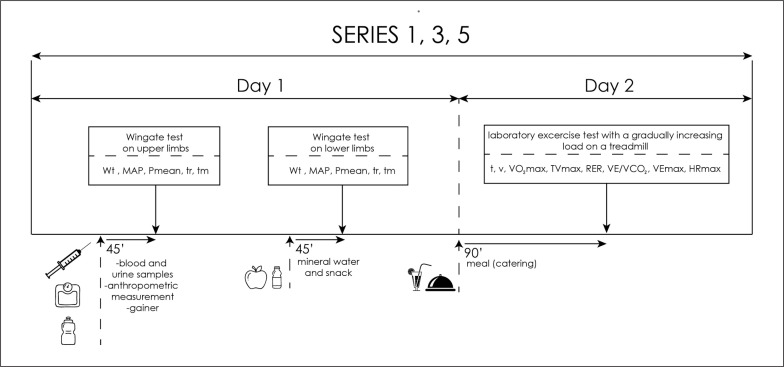
Scheme of anaerobic and aerobic test days. Abbreviations: Wt – total work performed; Pmean – mean anaerobic power; MAP – maximum anaerobic power; t_r_ – time of reaching maximum power; t_m_ – time of maintaining maximum powe; t-time; v- speed; VO_2_max- maximum oxygen uptake; TVmax- maximum tidal volume; RER- respiratory exchange ratio; VE∙VCO_2_^-1^ - respiratory equivalent carbon dioxide; V_E_ max- maximum minute ventilation; HRmax- maximum heart rate

After the blood was drawn, the participants consumed a standardized meal. For each participant of the experiment, a carbohydrate-protein supplement was prepared for consumption (a gainer made of hydrolysed beef protein without milk proteins). Moreover, each of the subjects consumed ad libitum highly mineralized mineral water. Then, the subjects started the exercise test to assess anaerobic capacity. Afterwards, they performed their first anaerobic test – Wingate’s 20-second test for upper limbs. The load was 4.5% of the body mass for the upper limbs (the Monark 834e, Sweden) and 7.5% of the body mass for the lower limbs (the Monark 894e cycle ergometer, Sweden). The software used (MCE 4.2, JBA Staniak, Poland) allowed the following indicators to be registered: total work performed (Wt [kJ], Wt [J ∙ kg^−1^]), mean anaerobic power (Pmean [W], Pmean [W ∙ kg^−1^], maximum anaerobic power (MAP [W], MAP [W ∙ kg^−1^]) and the time of reaching (t_r_ [s]) and maintaining (t_m_ [s]) maximum power. After the first anaerobic test, the players consumed a snack in form of a large apple (approx. 150 kcal) and hydrated themselves with highly mineralized water ad libitum. The competitors rested passively between the tests, and after 45 min the subjects began the Wingate test for lower limbs. On the second day, 1.5 hours before starting the aerobic test the subjects ate a meal planned in the food ration (catering) or its liquid substitute (gainer) and then they performed a laboratory exercise test with a gradually increasing load on a treadmill. To determine the maximum oxygen uptake (VO_2_max), using the direct method and the maximum heart rate (HRmax), a laboratory test “until failure” was carried out with a gradually increasing load on a treadmill (h/p/Cosmos Saturn treadmill, Germany). The heart rate (HR) was monitored every 30 seconds during the entire exercise test using the Polar S-410 cardiac monitor (Polar-Electro, Finland). The exercise test was preceded by a 2-minute recording of the initial values of cardiovascular and respiratory indicators on the treadmill (in a standing position). The test started with a 6-minute run at the speed [v] of 7 km/h, the same for all test subjects. Then, running speed was increased by 1.2 km ∙ h^–1^ every 2 min. The trial was performed until the athlete reported volitional exhaustion and refused to continue the test. Selected indicators of the respiratory and circulatory systems were analysed, including: maximum oxygen uptake (VO_2_max [l/min and ml/min/kg]), maximum minute ventilation (VEmax [l/min]), maximum tidal volume (TVmax), respiratory exchange ratio (RER), respiratory equivalent carbon dioxide (VE ∙ VCO_2_^−1^), heart rate (HR [bpm]). The level of indicators was recorded by the Cortex Metalyzer 3B ergospirometer (Germany) and using the MetaSoft Studio computer program (Germany) (Figure 2).

### Statistical analysis

The MS Excel 2010 spreadsheet and the PQStat statistical package, version 1.8.0.338, were used for the statistical analysis of the collected results and their interpretation. The basic characteristics of the examined variables were calculated, i.e.: median (Me), upper quartile (Q1), lower quartile (Q3), delta, arithmetic mean (X), standard deviation (SD) and the minimum (min) and maximum (max). The distribution of data was different from normal, so the results of the analysed scales between the groups PD and CD were compared with the non-parametric Mann-Whitney U test in series 1, 2, 3, 4, 5 for components of body composition and haematological parameters in fasting blood and urine biochemistry or in 1, 3, 5 for indicators of aerobic and anaerobic capacity. Intra-group changes in particular series were compared with Friedman’s test and post-hoc Dunn’s test with Bonferroni’s correction. The test probability at the level of *p <* 0.05 was assumed as significant and at the level of *p <* 0.01 was assumed as highly significant.

## RESULTS

### Analysis of changes in body mass and body composition (muscle mass, adipose fat mass, bone density, content of minerals and hydration level) during the experiment.

There were no significant differences between groups in particular series during the experiment, in all anthropometric parameters and body structure components. During the experiment, significant decreases in BM, BMI, and FM as well as an increase in the FFM (%, but not in kg) in both groups in the last series were observed (Table 1). At the end of the experiment, body mass significantly decreased (*p* < 0.01) in both groups compared to the initial value: in PD by 2.91 kg, and in the CD by 2.95 kg, with the largest decrease (4.45 kg) recorded after 6 weeks in the experimental group. In both groups at the end, significant decreases in BMI (*p* < 0.01) (-0.93 kg/m^2^ in PD and -0.5 kg/m^2^ in CD) were also observed. In the PD, between the beginning and the end of the experiment, there was a reduction in fat mass of 5% (*p* < 0.001), and in the CD of 1% (*p* = 0.01). Similar observations were made for the analysis of the fat mass in kg. In the PD, FFM increases by 3.6% (*p* < 0.001) were observed at the end of the experiment compared to the baseline value, and in the CD by 1.89% (*p* = 0.02). Analysis of changes in FFM in kg showed a slight non-statistically significant decrease, in both groups during the experiment (Table 1).

**TABLE 1:** Anthropometric parameters, body structure components, haematological and biochemical blood values. Data are reported as median, quartiles and point of change (absolute changes).

	PD (n = 9)						CD (n = 6)					
	1 Me (Q1; Q3) 1–2		1–3	1–4	1–5	F	1 Me (Q1; Q3) 1–2		1–3	1–4	1–5	F
Anthropometric parameters												
BM [kg]	91.36 (87.50;99.50)	-1.76	-0.19*	-4.45**	-2.91**	**	88.40 (84.70;100.65)	-0.8	-0.85	-2.3	-2.95**	**
BMI [kg/m^2^]	26.07 (25.75; 27.01)	-0.82	-0.85*	-0.64**	-0.93**	**	25.99 (25.90; 27.90)	-0.28	-0.31	-0.49	-0.5**	**
FM [%]	22.60 (19.00; 23.30)	-1.6	-3.0**	-3.20**	-5.0**	**	19.15 (18.45; 24.28)	-1.5	-2.1	-2.25**	-1.0*	**
FM [kg]	18.90 (16.15; 21.55)	-1.67	-2.03	-2.73**	-3.45**	**	16.17 (13.89; 23.41)	-1.41	-1.9	-2.3**	-2.11**	**
FFM [%]	78.39 (73.95; 81.85)	+1.18	+2.61*	+3.03**	+3.6**	**	81.68 (76.77; 82.38)	+1.48	+2.08	+2.17**	+1.89*	**
FFM [kg]	72. 93 (67.49; 75.74)	-0.04	-1.77	-1.79	-1.16	NS	72.47 (70.84; 77.33)	+0.69	+0.86	+0.74	+0.72	NS
MM [%]	42.03 (39.00; 42.87)	-0.69	-1.06*	-0.44*	+0.6	*	43.17 (42.00; 43.57)	-2.14	-1.67	-0.65	-1.05	NS
MM [kg]	38.20 (35.11; 41.13)	-1.71	-1.73*	-1.43*	-0.75	*	38.18 (36.72; 42.41)	-1.04	-1.5	-1.18	-1.0	*
BMC [g]	3877.00 (3548; 4207)	-54	-48	-60	-52	*	3972 (3741; 4229)	+27	+1	+11	-2	NS
BMD [g/cm^2^]	1.42 (1.39; 1.58)	-0.02	-0.03	+0.02	+0.01	**	1.55 (1.44; 1.63)	-0.07	-0.07	-0.08	-0.05	NS
Blood counts												
HGB [g/dl]	15.50 (14.80; 16.00)	-0.5	-0.4	0	-0.4	NS	15.50 (14.88; 15.68)	-0.2	+0.1	+01	0	NS
HCT [%]	45.80 (43.2; 47.60)	-1.3	-1.0	-0.7	-1.0	NS	45.60 (43.73; 46.35)	-1.0	+0.1	-0.55	-0.95	NS
Erythrocytes [mln/µl]	5.44 (5.20; 5.53)	-0.13	-0.11	-0.15	-0.29	NS	5.22 (5.07; 5.39)	+0.02	+0.05	+0.14	-0.16	NS
MCHC [g/dl]	34.30 (33.50; 34.30)	0	-0.40	+0.2	+0.1	NS	34.00 (33.93; 34.15)	-0.05	+0.25	+0.20	+0.60	NS
MCH [p/g]	29.16 (28.60; 29.80)	+0.14	+0.14	+0.14	-0.06	NS	29.35 (28.68; 30.40)	+0.40	+0.45	+0.25	+0.30	NS
MCV [fl]	84.34 (84.10; 87.50)	+0.16	-0.24	+0.16	+0.16	NS	86.95 (83.50; 89.95)	+0.01	+0.25	-0.75	-1.40	*
RDW-CW [%]	12.60 (12.20; 12.90)	-0.1	-0.1	-0.2**	-0.2	*	12.70 (12.25; 13.15)	+0.05	+0.2	-0.1	-0.3	NS
Leukocytes [tys/µl]	6.79 (6.44; 7.78)	-1.5	-1.46#	-1.09	-0.58	**	8.14 (6.03; 8.67)	-1.34	-1.51#	-1.83	-1.78	NS
Monocytes [tys/µl]	0.60 (0.50; 0.60)	-0.10	-0.10#	-0.10#	0	**	0.65 (0.53; 0.93)	0	+0.05#	-0.05#	-0.05	NS
Lymphocytes [tys/µl]	2.80 (2.40; 3.43)	-0.4	-0.5**	-0.1	-0.5	**	2.70 (2.25; 3.15)	-0.5	-0.3	-0.45	-0.6	NS
Neutrophils [tys/µl]	3.60 (3.20; 4.00)	-0.9	-1.1**	-0.8	-0.8	**	3.95 (3.08; 4.30)	-0.35	-0.5	-0.9	-1.25	NS
Basophils [tys/µl]	0.03 (0.03; 0.05)	0	+0.02	-0.01	-0.01	NS	0.065 (0.04; 0.08)	0	-0.005	-0.005	-0.005	NS
Eosinophils [tys/µl]	0.11 (0.15; 0.33)	+0.03	+0.08	+0.07	+0.07	NS	0.17 (0.13; 0.29)	-0.03	-0.05	-0.01	+0.05	NS
Platelets [tys/µl]	264 (249.00; 277.10)	-2	-18	-30	0	*	259.5 (252.5; 321.25)	+24	+1	+6.5	+5.5	NS
PDW [fl]	13.50 (12.58; 15.50)	+1.3	+0.9	+1.5	+0.2	*	14.55 (13.40; 15.33)	+0.85	+1.6	+1.05	+1.4	NS
P-LCR [%]	36.00 (32.19; 41.70)	+1.7	+3.0	+4.0	-0.30	*	37.60 (34.53; 40.75)	+4.95	+4.35	+3.6	+2.70	*
MPV [fl]	11.20 (10.78; 12.10)	+0.4	+0.4	+0.6	+0.1	*	11.45 (11.10; 11.80)	+0.55	+0.55*	+0.50	+0.35	*
PCT [%]	0.3 (0.3; 0.3)	0	0	0	0	NS	0.3 (0.3; 0.375)	0	0	0	0	NS
Lipid profile												
TC [mmol/l]	3.84 (3.77; 4.31)	+0.03	+0.53	+0.33	+0.27	NS	4.65 (4.22; 4.77)	-0.41*	-0.10	-0.27	-0.19	*
HDL-C [mmol/l]	1.39 (1.31; 1.91)	+0.15	0	+0.03	+0.24#	NS	1.29 (1.08; 1.64)	-0.10	-0.11	-0.09	-0.06#	NS
LDL-C [mmol/l]	2.18 (2.05; 2.52)	-0.25	+0.27	-0.06	+0.05	NS	2.14 (1.81; 2.54)	-0.07	+0.30	+0.28	+0.36	NS
TG [mmol/dl]	1.02 (0.78; 1.38)	-0.31*	-0.29	-0.37	-0.11	*	1.36 (1.16; 1.96)	-0.48	+0.5	-0.11	-0.35	NS
Carbohydrates and fat metabolism												
Glucose [mmol/l]	5.29 (4.86; 5.44)	-0.6*	-0.45	-0.69**	-0.13	**	5.10 (5.04; 5.19)	-0.29	-0.31	-0.15	-0.09	NS
Insulin [µIU/ml]	7.20 (6.69; 8.20)	-1.3	-1.5	-1.65	-0.24	NS	6.63 (5.70; 7.51)	-0.48	+0.86	+0.87	-0.58	NS
FFA [mmol/l]	0.56 (0.24; 0.68)	-0.07	-0.24	-0.18	-0.27	NS	0.24 (0.14; 0.39)	+0.32	+0.21	+0.04	+0.05	NS
b-HB [mmol/l]	0.05 (0.03; 0.06)	+0.14	+0.05	+0.09	+0.03	NS	0.04 (0.03; 0.04)	+0.12	+0.10	-0.02	+0.03	NS
Electrolytes												
Sodium [mmol/l]	142.00 (141.00;95.00)	-1	0	-2	+13*	*	141.5 (139.50;142)	-2.5	-0.5	-1	0	NS
Calcium [mmol/l]	2.36 (2.29; 2.45)	+0.09	+0.13**	+0.11*	+0.14*	**	2.42 (2.39; 2.47)	+0.06	+0.10	+0.02	-0.01	NS
Magnesium [mmol/l]	0.83 (0.81; 0.84)	+0.06	+0.03	+0.05	+0.02	NS	0.83 (0.81; 0.85)	+0.11*	+0.06	+0.03	0	**
Potassium [mmol/l]	4.51 (4.34; 4.79)	0	-0.01	+0.11	-0.14	NS	4.58 (4.36; 4.92)	-0.03	+0.02	+0.09	+0.14	NS
Kidney parameters												
Creatinine [µmol/l]	93.00 (81.00; 95.00)	+13	+12	+10*	+1	*	94.00 (93.25; 94.00)	+6.5	+7	+1.5	+9.5*	**
eGFR [ml/min/1.73m^2^]	88.13 (83.79; 91.00)	-12.32	-11.71	-9.94	-1.09	*	85.93 (84.09; 86.19)	-6.9	-8.36	-3.53	-9.63	*
Urea [mmol/l]	5.00 (4.60; 8.49)	+2.2	+1.6	+1.9	+1.9	NS	5.70 (5.53; 6.10)	-0.35	+0.4	+0.55	+1.7	NS
Uric acid [µmol/l]	320 (310.00; 368.00)	+51	+18	+42	+19	NS	303(274.25; 314.50)	+71*	+44	+36	+34	NS
Cystatin C [mg/dl]	0.86 (0.76; 0.86)	-0.08	-0.08	-0.02	+0.07	*	0.79 (0.70; 0.89)	+0.04	+0.07	+0.03	+0.10	**
Urine pH	6.5 (5; 7)	-1.5	-1.5	-1.5	-1.5	NS	5.5 (5; 6.75)	0	-0.5	+0.25	-0.5	NS
Liver parameters												
ALT [U/l]	21.00 (18.00; 32.00)	+5	+4.7	+1	+1	NS	25.50 (19.25; 34.75)	-1.6	-3.7	-4.2	-4.9	NS
AST [U/l]	23.00 (19.00; 28.00)	+1	+1	-2.2	-1.2	NS	26.50 (25.25; 27.75)	+1.52	-1.82	-4.12	-3.07	NS
Alkaline Phosphatase [U/l]	88.00 (63.00; 106.00)	-9	-12	-8	+3	*	77.50 (67.50; 90.50)	+4.5	+4	-1.5	0	NS
GGTP [mmol/l]	17.00 (11.00; 20.00)	-2	-4	-4*	-4	*	16.00 (11.75; 20.25)	-2	-3	-3	-2.5	NS
Total bilirubin [µmol/l]	7.70 (6.10; 8.50)	+1.5	+2.2	+2.8	0	NS	7.40 (5.78; 12.25)	+2.75	+3.2	+1.55	+1.55	NS

Abbreviations: PD – Paleo diet; CD – control diet; Me – median; Q1 – upper quartile; Q3 – lower quartile; 1–2; 1–3; 1–4; 1–5 – differences between particular series; F – Friedman’s Test; BM – body mass; BMI – body mass index; FM – fat mass; FFM – fat free mass; MM – muscle mass; BMC – body mineral content; BMD – body mass density; HGB – haemoglobin; HCT – hematocrit; MCHC – mean corpuscular hemoglobin concentration; MCH – mean corpuscular haemoglobin; MCV – mean corpuscular volume; RDW-CV – red blood cell distribution with coefficient of variation; PDW – platelet distribution width; P-LCR – platelet-large cell ratio; MPV – mean platelet volume; PCT – procalcitonin; TC – total cholesterol; HDL-C – high-density lipoprotein cholesterol; LDL-C – low-density lipoprotein cholesterol; TG – triglycerides; FFA – free fatty acids; b-HB – beta-hydroxybutyrate; e-GFR – estimated glomerular filtration rate; ALT – alanine aminotransferase; AST – aspartate aminotransferase; GGTP – gamma-glutamyl transferase; italics – changes between 1–5 series;

* – test probability at the level of p < 0.05 intragroup;

** – p < 0.01;

# – test probability at the level of p < 0.05 intergroup; ## – p < 0.01 in U-Mann test.

### Analysis of changes in selected haematological and biochemical indicators of blood and urine during the experiment

There were no significant differences between groups in particular series during the experiment in all haematological and biochemical indicators of blood and urine (Table 1). Only HDL-C was significantly higher (*p* = 0.039) in the last series in the PD compared to the CD (1.63 mmol/l vs. 1.23 mmol/l). All the values were within the reference range during the experiment. Intergroup differences were related to lower amounts of monocytes and leukocytes in the middle of the experiment in the group of athletes on a PD, with the differences levelling off at the end of the experiment, and they were similar or the same as at the beginning of the experiment. No significant intergroup differences in lipid profile, carbohydrates and fat metabolism, kidney and liver parameters, or electrolytes were observed between series 1–5 except for sodium and calcium in the PD, which increased significantly (*p* < 0.05) at the end of the experiment (Table 1). All urinary indicators stayed at reference values during the interventions. Urinary ketones were absent or traced in both groups, and the differences were not significant, either between groups or within groups during the experiment.

### Analysis of the level of anaerobic and aerobic performance of handball players during the experiment

There were no significant differences between groups in particular series during the interventions in all the values of anaerobic capacity (Table 2). In the Wingate test on upper limbs, there were only single intragroup changes, consisting of a significant decrease in the Wt (kJ), MAP (W) and Pmean (W) in the experimental group. A significant decrease (*p* = 0.014) in the total amount of work performed between the beginning and the end of the intervention (10.31 kJ vs. 9.89 kJ) was observed. Also, in MAP and Pmean significantly lower values were found between the initial and final series: 597.93 W vs. 564.00 W and 515.71 W vs. 495.00 W, respectively (Table 2). In the Wingate test on lower limbs in the PD, post-hoc analysis showed a significant decrease (*p* = 0.001) between the beginning and the end of the intervention in the Wt (17.65 kJ vs. 15.85 kJ). Similarly, the total work performed per kilogram of BM differed significantly (*p* = 0.004) in the PD. During the experiment significantly lower MAP values between the baseline and end series in the PD (*p* = 0.003) (1063.00 W vs. 946.00 W) and in the CD (*p* = 0.028) (1092.00 W vs. 1005.00 W) were observed. A significant decrease (*p* = 0.018) in MAP per kg BM was observed between the mid point and the end of the experiment (11.47 W ∙ kg^−1^ vs. 10.91 W ∙ kg^−1^) only in the PD. During the intervention, significant differences (*p* = 0.008) in Pmean with a downward trend (*p* = 0.002) were observed only in the PD. These changes consisted in significantly lower (*p* = 0.007) mean anaerobic power, especially between the beginning and end of the experiment (882.00 W vs. 792.00 W) (Table 2). There were no significant differences between the groups in individual series or intragroup differences during the experiment, determined by the VO_2_max (minute and relative to BM), VEmax, VE ∙ VCO_2_^−1^, RER, and the time of the test with a gradually increasing load on a treadmill, except for a significant decrease (*p* = 0.018) of TVmax, between the beginning and the end of the intervention (*p* = 0.014) (2.85 l vs. 2.73 l). HRmax significantly decreased between the initial and final series in the PD (*p* = 0.001) (187 bpm vs. 184 bpm) and CD (*p* = 0.014) (187 bpm vs. 183 bpm) group (Table 2). Tables with the results showing arithmetic means and standard deviation are included in the paper’s appendix.

**TABLE 2:** Anaerobic and aerobic capacity. Data are reported as median, quartiles and point of change (absolute changes).

	PD (n = 9)				CD (n = 6)			

	1 Me (Q1; Q3)	1–3	1–5	F	1 Me (Q1; Q3)	1–3	1–5	F
Wingate test on upper limbs								
Wt [kJ]	10.31 (10.13; 11.20)	+0.26	-0.42*	*	10.27 (9.61; 11.00)	+0.27	-0.21	NS
Wt [J ∙ kg^−1^]	112.00 (107.00;117.00)	-4	+4	NS	110.00 (104.50; 117.75)	+5	+7.5	NS
MAP [W]	597.93 (586.00; 660)	+7.07	-33.93**	*	597.50 (560.00; 664.25)	+11.5	-15.5	NS
MAP [W ∙ kg^−1^]	6.45 (5.96; 6.99)	-0.17	+0.06	NS	6.41 (6.23; 6.75)	+0.43	+0.44	NS
Pmean [W]	515.71 (506.00; 560.00)	+12.29	-20.71*	**	513.00 (480; 550)	+14	-10.5	NS
Pmean [W ∙ kg^−1^]	5.60 (5.30; 5.90)	-0.20	+0.20	NS	5.50 (5.23; 5.85)	+0.25	+0.35	NS
t_r_ [s]	5.14 (5.10; 5.23)	+0.18	+0.4	NS	4.71 (4.30; 5.40)	+0.03	+0.32	NS
t_mz_ [s]	3.70 (3.16; 3.75)	+0.32	-0.07	NS	2.52 (2.34; 2.62)	+0.87	+0.96	NS

Wingate test on lower limbs								
Wt[kJ]	17.65 (17.27; 19.71)	-1.02	-1.8**	**	17.40 (16.45; 17.83)	-0.05	-2.53	NS
Wt [J ∙ kg^−1^]	191.21 (181.00; 196.00)	+2.79	-12.21**	**	186.00 (182.25; 191.25)	+5.36	-5.36	NS
MAP [W]	1063.00 (1028.00; 1164.15)	-5	-117**	**	1092.50 (954.75; 1141)	-29	-87.5*	*
MAP [W ∙ kg^−1^]	11.47 (10.62; 11.65)	0	-0.56	*	11.50 (10.58; 12.68)	-0.18	-0.55	NS
Pmean [W]	882.00 (864.00; 985;00)	-50	-90**	**	870.00 (822.51; 892.00)	-2.5	-38.5	NS
Pmean [W ∙ kg^−1^]	9.55 (9.10; 9.80)	+0.15	-0.65	*	9.30 (9.08; 9.60)	+0.27	-0.11	NS
t_r_ [s]	4.08 (3.86; 4.23)	+0.02	+0.25	NS	3.63 (3.25; 4.44)	-0.09	-0.10	NS
t_m_ [s]	3.03 (2.65; 3.73)	-0.13	-0.28	NS	2.52 (2.34; 2.62)	+0.22	+0.3	NS

Aerobic capacity test								
VO_2_max [l/min]	4.40 (3.85; 4.64)	-0.10	-0.11	NS	4.26 (4.09; 4.43)	-0.18	-0.05	NS
VO_2_max [ml/kg/min]	46.00 (42.33; 49.00)	+1	0	NS	48.00 (43.25; 49.00)	+0.93	+0.68	NS
VEmax [l/min]	147.60 (143.60; 166.70)	+5.5	-0.7	NS	153.55 (146.28; 162.78)	+3.1	-0.65	NS
TVmax [l]	2.85 (2.65; 3.38)	0	-0.08*	*	2.69 (2.61; 2.90)	+0.11	+0.09	NS
RER	1.10 (1.08; 1.13)	0	+0.02	NS	1.14 (1.12; 1.17)	+0.03	+0.01	NS
VE ∙ VCO_2_^−1^	29.80 (28.60; 32.80)	-0.70	-0.80	NS	29.70 (29.23; 30.70)	+0.45	-0.79	NS
HRmax [bpm]	187 (184; 195)	0	-3**	**	187 (176; 197)	-1	-4*	*
t [min]	16.50 (15.00:18.50)	+0.5	0	NS	17.75 (15.88;18.88)	+1.25	+0.5	NS
v [km/h]	14.86 (14.10; 15.50)	+0.54	+0.54	*	16.05 (14.53; 16.60)	+0.7	+0.6	NS

Abbreviations: PD – Paleo diet; CD – control diet; Me – median; Q1 – upper quartile; Q3 – lower quartile; 1–3; 1–5 – differences between particular series; Wt– total work performed; MAP – maximum anaerobic power; Pmean – mean anaerobic power; t_r_– time of reaching maximum power; t_m_ – time of maintaining maximum power; VO_2_max – maximum oxygen uptake; VE_max_ – maximum minute ventilation; TVmax – maximum tidal volume; RER – respiratory exchange ratio; VE ∙ VCO_2_^−1^ – respiratory equivalent carbon dioxide; HRmax – maximum heart rate; t-time; v – speed; F – Friedman’s Test; italics – changes between 1–5 series;

* – test probability at the level of *p <* 0.05 intragroup;

** – *p <* 0.01.

## DISCUSSION

There is no research assessing the impact of the Paleo diet on body composition in a group of professional athletes. In our research the eight-week application of the Paleo and control diet had a positive effect on body composition through a decrease in BM, BMI, FM (% and kg) and an increase in FFM% (but not in kg). The beneficial impact of PD on body structure (BM, waist circumference, weight/height ratio and % of FM) applies to various groups: healthy people with a sedentary lifestyle [9], inactive people with planned physical activity (aerobic and resistance training) [4–6] or patients with: ischemic heart disease [2], lipid profile disorders [3], overweight and obesity [4, 5], diabetes [5, 6], or metabolic syndrome [7]. The applied Paleo diets were characterized by consumption ad libitum, while with the normoenergetic nature of PD, the changes showed mainly no body mass reduction effect [8] but were often related to the improvement of some metabolic indicators [7, 8]. There are only a few studies in which the normoenergetic model of the Paleo diet was used [7, 8]. It is not unequivocally determined whether applying the energy deficit or the distribution of macronutrients is the cause of the changes in body weight. In the study by Michalczyk et al. [11], FM of basketball players did not depend on the amount of fat, but the amount and quality of carbohydrates consumed in the Carbo-L (a 7-day carbohydrate loading protocol) or low-carbohydrate diet and the increase in FM occurred only as a result of loading with carbohydrates. Conversely, a meta-analysis of 94 studies on lowcarbohydrate diets found that weight loss following a low-carbohydrate diet was associated with a reduction in energy consumption and dietary duration, but not with a reduction in dietary carbohydrates [16]. Similarly, we observed a significant reduction in BM and FM in both the experimental and control groups, although in the first weeks the reduction effect was more visible in the PD group. Moreover, maintaining FFM during fat reduction was observed in athletes and inactive people using a lower carbohydrate (LCD) and ketogenic diet (VLCD-KD) [17–18]. In contrast, McSwiney et al. [18] found that after a short-term ketogenic diet in endurance-trained males there was a decrease in both FM and FFM. It seems that the PD (as well as the KD and LCD), when it provides an adequate supply of calories and is accompanied by properly selected training, should result in the maintenance of FFM, especially in a short time of use.

An important factor in the discussion on the necessity of following alternative diets is their impact on health. Scientists dealing with alternative strategies mention a number of possible positive consequences of adopting various nutritional strategies, e.g., the ketogenic diet, training with high or low availability of carbohydrates [19, 20]. It is recommended to implement dietary strategies to increase antioxidant potential of the diet (e.g. fresh fruits, vegetables, sprouts as well as seeds) [21]. In addition, reduction in consumption of refined foods, including carbohydrates, may be critical in preventing or treating metabolic-related cognitive deficits [22]. We found that the eight-week use of the PD does not cause significant, unfavourable changes in health. We observed no effect on lipid profile, except for higher HDL cholesterol concentration after eight weeks of using the Paleo diet. This trend is in line with analyses of the use of the Paleo diet in inactive people. In a meta-analysis Ghaedi et al. [23] found that PD reduces the concentration of total cholesterol (TC), LDL cholesterol and triglycerides (TG) and increases the concentration of HDL cholesterol. Similar results were obtained by Manheimer et al. [24], observing favourable changes in HDL concentration. According to Noakes et al. [25], changes in the blood lipid profile depend on the dietary fat content. A positive effect on the lipid profile in the group of athletes on the LCD and KD was also observed and according to the authors it may have been caused by the predominance of polyunsaturated fatty acids [11, 26]. Moreover, the Paleo diet provided significantly more pro-inflammatory saturated fatty acids (SFAs) and cholesterol. Unfavourable changes in lipid profile that result from applying the high fat diets are more pronounced after diets high in SFAs. Higher consumption of SFA and cholesterol could cause hypercholesterolaemia in 20 ultra-endurance athletes [27] and low-carbohydrate diets promote atherosclerotic changes in athletes [28]. The Paleo diet is not a risk that stimulates the development of cardiovascular diseases resulting from disturbances in the lipid profile [23]. Therefore, it can be concluded that with a high supply of polyunsaturated fatty acids (PUFAs) even a high content of SFAs and cholesterol in the diet did not have a negative effect on the lipid profile. In our research, no changes in all assessed indicators of metabolism of carbohydrates and fat were found, which remained within the reference values throughout the entire experiment in the PD and CD, as also occurred in the group of basketball players on an LCD [11]. A meta-analysis by Jamka et al. [29] showed that the Paleo diet may improve glucose tolerance, reduce insulin secretion, and increase insulin sensitivity in people with impaired glucose metabolism, as well as other healthy diets (the Mediterranean diet, diabetes diet, and a diet recommended by the Dutch Health Council). Other studies with limited carbohydrate intake showed a decrease in blood insulin concentration at rest or during exercise [30, 31] or no effect [26, 32–34]. Scientific studies show that lipolysis and fat oxidation increased as insulin levels decreased [25]. Elevated ketone levels are necessary to induce decreased insulin production and thus increase fat utilization [35]. Perhaps that is why in our research no decrease in blood insulin was observed. In numerous studies using the KD, the level of ketosis considered desirable was also not observed [36]. In turn, the studies by Zając et al. [26] showed that the KD in cyclists caused a fourfold increase in the concentration of b-HB before exercise and a twofold increase in resting FFA concentration in plasma. Our analysis did not show that use of the PD increased the oxidation of fatty acids, and the level of ketones in the urine was minimal or undetectable in the course of the experiment.

There was no effect of the 8-week PD on blood counts, electrolytes and kidney and liver function indicators, which remained within the reference intervals throughout the intervention. In the research by Urbain et al. [30], no effect of the 6-week ketogenic diet on the level of total bilirubin, AST, or creatine was found, but a significant decrease in alkaline phosphatase, and increases in ALT, concentration of urea and uric acid were observed at the end of the experiment in the group of 42 healthy adults. Uric acid concentration may serve as one biomarker of keto-adaptation in the kidneys [37]. Researchers have found that serum uric acid remains above baseline levels even after 12 weeks on the KD [38]. In our research, no changes in uric acid concentration in the PD were found. On the other hand, long-term use of an acidic diet in combination with exercise leads to an initial impairment of renal function [39]. In the analysis of the effect of the 6-week ketogenic diet on healthy adults, no changes in creatinine levels were found.

Numerous studies have been conducted investigating the potential ergogenic effects of low-carbohydrate diets. The LCD accelerate the feeling of fatigue and reduce concentration by reducing the effectiveness of training [28]. On the other hand, many studies have shown that LCD (most often with higher protein content, above 30%) through weight loss, and in some cases resulting in an increase in FFM, especially in weight-category sports, may contribute to the improvement of exercise capacity [17, 18, 34, 40]. Especially in short-term resistance efforts, it is recommended to eat more carbohydrates, which is based on evidence supporting the importance of glycogen as both an energy “fuel” and a regulator of skeletal muscle adaptive responses during exercise [41]. On the other hand, there is insufficient evidence that carbohydrates are required for mammalian target of rapamycin complex-1 (mTORC-1) signalling during muscle protein synthesis [42], especially when dietary protein intake is adequate [43]. Thus, adaptive changes resulting from the use of LCD may, on the one hand, improve the aerobic capacity, but on the other hand may reduce the possibility of using exogenous carbohydrates, which in turn may contribute to the lack of improvement in efficiency, and may even worsen the exercise capacity, during more intense physical efforts [19, 44]. An undoubted advantage of the Paleo diet is the sufficient carbohydrate content (in the context of glycogen availability), but the disadvantage may be the lack of ketosis occurring in the KD – enabling the use of fatty acids in energy processes. However, as previously demonstrated in some sports, for instance team sports, it is not necessary to consume high carbohydrates in the daily diet due to the lower training volume and the shorter duration of the competition [11]. In this case, the Paleo diet may be the correct choice. It has been reported that supervised exercise in combination with the Paleo diet (PDEX) influenced the maintenance of LBM in men and increased cardiovascular capacity in patients with diabetes [4]. Higher VO_2_max values have also been observed in healthy, inactive, non-overweight people after PD. However, these changes would be the result of training rather than diet [9]. In the research of Popp et al. [45], which compared the PD in combination with physical activity to a “MyPlate” diet that included both aerobic training and resistance training, a greater improvement in exercise capacity was observed with a rational diet (“MyPlate”). Kang et al. [36] identified a few independent studies in which no effect of a KD on VO_2_max was demonstrated [32–34, 46, 47], while in two research papers, the authors reported a decrease in VO_2_max in l/min [30, 48], and an increase in VO_2_max [26]. A significant increase in VO_2_max was observed in a group of cyclists using KD compared to a mixed diet [26]. The authors explain the improvement in VO_2_max (ml/kg/min) accompanying the use of the KD as a consequence of a decrease in BM and FM [26]. Our research did not show an effect of the use of the PD on the maximum oxygen uptake or other indicators of aerobic capacity, either in absolute or relative values, despite the BM reduction. So, it was found in our research that the normoenergetic PD model can be successfully used by athletes without adversely affecting aerobic capacity. Analysing the indicators of anaerobic capacity, we observed a decrease in total amount of work performed in the PD, the maximum and mean power in absolute values in both the Wingate test and in relative Wt [J ∙ kg^−1^] only in the Wingate test on lower limbs. However, the time to achieve and maintain MAP in the PD did not decrease significantly. Kang et al. [36] reported seven studies assessing the effect of the KD on anaerobic capacity. In a few, there was no effect on muscle strength and power [17, 49, 50]. In studies by Rhyu and Cho [40] in both diet groups, KD and NKD (non-ketogenic diet), with a distribution of macronutrients similar to the PD, the diet resulted in a decrease in MAP and Pmean of taekwondo athletes. Research suggests that low-carbohydrate, high-fat or ketogenic diets may be useful in weight loss, most often without compromising strength or power [36]. It cannot be clearly stated that the Paleo diet adversely affects anaerobic capacity in athletes. A longer follow-up and larger sample size are recommended in future clinical trials of the Paleo diet used on athletes.

## LIMITATIONS

The factor limiting the research was the size of the research group. The high cost of catering to meet individual energy needs, representing a high level of sports and the specific products of the Paleo diet (meat, fish, seafood, eggs, nuts, fruit, and vegetables) made it impossible to include more athletes in the experiment (22 people were planned and 15 athletes completed the study). Preparing meals with the assumption of maintaining balanced energy especially with high energy demand and the specific composition of the Paleo diet eliminated the possibility of methodological errors. The presented results of the pilot studies are the nucleus of the planned research process on a larger scale.

## CONCLUSIONS

1. Eight-week intervention of the Paleo diet resulted in a decrease in body mass, body mass index and the amount of fat mass, which should be considered a beneficial effect of the diet in the context of the body structure of handball players.
2. There was no adverse effect of the Paleo diet on the health status of research participants, assessed with the following indicators: peripheral blood count, lipid profile, carbohydrate and fat metabolism, concentration of selected electrolytes, kidney and liver function and urine assessment. The use of the Paleo diet significantly increased the concentration of HDL cholesterol in the group following the Paleo diet compared to the rational diet, even with a significantly higher content of SFA and cholesterol in the Paleo diet (3 and 2 times higher than the norm, respectively).
3. Eight-week use of the Paleo diet slightly adversely affects anaerobic capacity, reducing some of the assessment indicators, i.e., total work performed, mean and maximum anaerobic power, and does not affect the level of aerobic capacity of handball players.
4. The Paleo diet can be used by representatives of disciplines that do not require high availability of carbohydrates (e.g., team sports) and players planning to obtain optimal body composition in a short period of time.

## Figures and Tables

**SUPPLEMENTARY TABLE 1:** Anthropometric parameters, body structure components, hematological and biochemical blood values. Data are reported as arithmetic mean, standard deviation and point of change (absolute changes).

	PD (n = 9)						CD (n = 6)					

	1x ± SD	1–2	1–3	1–4	1–5	F	1x ± SD	1–2	1–3	1–4	1–5	F
Anthropometric parameters												
BM [kg]	93.52 ± 9.74	-2.46	-3.47*	-4.47**	-4.65**	**	92.47 ± 13.10	-0.67	-1.05	-1.69	-2.45**	**
BMI [kg/m^2^]	26.73 ± 1.79	-0.71	-1.00*	-1.30**	-1.33**	**	27.16 ± 2.28	-0.22	-0.34	-0.52	-0.75**	**
FM [%]	23.25 ± 5.82	-1.73	-2.64**	-2.97**	-3.92**	**	21.68 ± 5.45	-1.73	-2.60	-3.31**	-3.53*	**
FM [kg]	20.70 ± 7.04	-1.71	-2.77	-3.25**	-4.09**	**	19.83 ± 8.04	-1.58	-2.42	-3.05**	-3.36**	**
FFM [%]	77.28 ± 5.82	+1.61	+2.63*	+2.76**	+3.69**	**	79.24 ± 5.26	+1.70	+2.56	+3.20**	+3.40*	**
FFM [kg]	72.06 ± 7.47	-0.41	-0.45	-0.94	-0.25	NS	72.63 ± 5.78	+0.94	+1.41	+1.37	+0.93	NS
MM [%]	41.10 ± 3.13	-0.88	-0.39*	-0.18*	+0.63	*	42.35 ± 1.70	-0.88	-0.75	+0.16	-0.16	NS
MM [kg]	38.37 ± 4.38	-1.81	-1.73*	-1.90*	-1.33	*	38.96 ± 4.03	-1.13	-0.89	-1.21	+3.39	*
BMC [g]	3877.29 ± 496.30	-14.56	-9.89	-4.48	-13.69	*	3997.00 ± 397.05	+3.33	-2.17	-10.33	-15.83	NS
BMD [g/cm^2^]	1.47 ± 0.12	-0.03	-0.04	0	-0.01	**	1.54 ± 0.11	-0.04	-0.05	-0.04	-0.03	NS

Blood counts												
HGB [g/dl]	15.31 ± 1.13	-0.34	-0.22	-0.12	-0.19	NS	15.28 ± 0.69	-0.21	+0.19	+0.17	-0.18	NS
HCT [%]	45.00 ± 3.21	-1.23	-0.89	-0.88	-1.24	NS	42.90 ± 1.89	-0.70	+0.50	+0.15	-1.32	NS
Erythrocytes [mln/*μ*l]	5.25 ± 0.46	-0.14	-0.08	-0.07	-0.08	NS	5.19 ± 0.33	-0.11	-0.02	0	-0.12	NS
MCHC [g/dl]	34.03 ± 0.57	+0.14	+0.20	+0.37	+0.56	NS	34.03 ± 0.44	+0.07	+0.04	+0.27	+0.60	NS
MCH [p/g]	29.24 ± 0.81	+0.08	-0.01	+0.12	-0.05	NS	29.48 ± 0.94	+0.25	+0.45	+0.35	+0.37	NS
MCV [fl]	85.95 ± 2.53	-0.11	-0.45	-0.64	-1.23	NS	86.63 ± 3.44	+0.57	+1.20	+0.42	-0.51	*
RDW-CW [%]	12.67 ± 0.42	-0.17	-0.21	-0.34**	-0.24	*	12.70 ± 0.44	+0.02	+0.02	+0.12	-0.18	NS
Leukocytes [tys/*μ*l]	7.33 ± 1.43	-1.58	-1.96#	-1.20	-1.00	**	7.74 ± 1.81	-0.91	-0.49#	-1.22	-0.88	NS
Monocytes [tys/*μ*l]	0.63 ± 0.16	-0.06	-0.15#	-0.13#	-0.01	**	0.73 ± 0.24	-0.08	-0.03#	-0.10#	-0.03	NS
Lymphocytes [tys/*μ*l]	2.95 ± 0.72	-0.61	-0.67**	-0.38	-0.47	**	2.77 ± 0.63	-0.42	-0.10	-0.10	-0.20	NS
Neutrophils [tys/*μ*l]	3.49 ± 0.86	-0.81	-1.10**	-0.62	-0.48	**	3.92 ± 1.59	-0.30	-0.24	-0.90	-0.60	NS
Basophils [tys/*μ*l]	0.05 ± 0.03	-0.01	0	-0.01	-0.01	NS	0.07 ± 0.03	-0.01	-0.01	-0.01	-0.01	NS
Eosinophils [tys/*μ*l]	0.24 ± 0.11	-0.06	-0.05	-0.01	-0.01	NS	0.23 ± 0.16	-0.06	-0.08	-0.04	+0.01	NS
Platelets [tys/*μ*l]	266.23 ± 31.93	-5.45	-21.00	-27.12	-6.23	*	290.33 ± 62.64	-7.00	-20.83	-6.16	-22.83	NS
PDW [fl]	14.14 ± 2.00	+0.05	+0.63	+0.99	-0.13	*	14.48 ± 1.17	+0.84	+1.25	+1.19	+1.32	NS
P-LCR [%]	35.64 ± 6.82	+1.69	+3.19	+3.72	-0.05	*	37.65 ± 4.09	+3.43	+3.85	+3.23	+2.65	*
MPV [fl]	11.24 ± 0.85	+0.29	+0.4	+0.49	+0.07	*	11.47 ± 0.48	+0.40	+0.50*	+0.44	+0.38	*
PCT [%]	0.30 ± 0	0	0	-0.01	0	NS	0.35 ± 0.08	-0.02	-0.03	-0.06	-0.05	NS

Lipid profile												
TC [mmol/l]	4.20 ± 0.76	-0.20	+0.30	+0.05	-0.07	NS	4.55 ± 0.63	-0.59*	-0.19	-0.23	-0.42	*
HDL-C [mmol/l]	1.49 ± 0.34	+0.20	-0.01	+0.02	+0.25#	NS	1.45 ± 0.48	-0.10	-0.08	-0.17	-0.11#	NS
LDL-C [mmol/l]	2.19 ± 0.61	-0.26	+0.20	+0.13	-0.01	NS	2.32 ± 0.72	-0.28	+0.12	+0.09	+0.01	NS
TG [mmol/dl]	1.17 ± 0.46	-0.34*	-0.34	-0.26	-0.19	*	1.70 ± 0.85	-0.43	-0.47	-0.32	-0.68	NS

Carbohydrates and fat metabolism												
Glucose [mmol/l]	5.20 ± 0.32	-0.59*	-0.31	-0.51**	-0.15	**	5.14 ± 0.18	-0.37	-0.28	-0.21	-0.17	NS
Insulin [*μ*IU/ml]	8.24 ± 3.35	-1.1	-1.55	-0.45	+0.01	NS	7.59 ± 2.85	+0.11	+1.72	+0.16	-1.62	NS
FFA [mmol/l]	0.50 ± 0.22	-0.01	-0.08	+0.07	-0.14	NS	0.34 ± 0.26	+0.25	+0.16	+0.04	0	NS
b-HB [mmol/l]	0.05 ± 0.02	+0.21	+0.12	+0.14	+0.09	NS	0.08 ± 0.11	+0.18	+0.06	+0.01	+0.03	NS

Electrolytes												
Sodium [mmol/l]	142.65 ± 2.02	-2.15	-1.35	-0.65	-0.21*	*	140.80 ± 1.34	-1.47	+0.2	+0.5	+0.70	NS
Calcium [mmol/l]	2.36 ± 0.08	+0.10	+0.15**	+0.12*	+0.11*	**	2.44 ± 0.07	+0.03	+0.07	0	+0.02	NS
Magnesium [mmol/l]	0.83 ± 0.03	+0.05	+0.05	+0.04	+0.02	NS	0.83 ± 0.04	+0.08*	+0.07	+0.03	-0.01	**
Potassium [mmol/l]	4.60 ± 0.28	-0.09	-0.11	+0.03	-0.07	NS	4.63 ± 0.29	-0.05	-0.01	-0.03	+0.06	NS

Kidney parameters												
Creatinine [*μ*mol/l]	90.26 ± 9.72	+8.07	+9.96	+9.41*	+2.96	*	93.33 ± 1.60	+11.00	+9.67	+4.84	+10.67*	**
eGFR [ml/min/1.73 m^2^]	86.40 ± 5.15	-6.59	-7.52	-6.62	-2.62	*	85.92 ± 2.61	-9.67	-9.03	-4.42	-9.94	*
Urea [mmol/l]	6.15 ± 1.90	+1.08	+0.63	+1.36	+0.38	NS	5.90 ± 1.22	-0.17	-0.20	+0.82	+1.07	NS
Uric acid [*μ*mol/l]	330.90 ± 47.06	+38.7	+34.46	+35.9	+5.21	NS	297.85 ± 49.16	+81.15*	+57.65	+58.95	+39.32	NS
Cystatin C [mg/dl]	0.82 ± 0.10	-0.04	-0.02	0	+0.06	*	0.79 ± 0.10	+0.04	+0.07	+0.04	+0.09	**
Urine pH	6.10 ± 0.92	-0.66	-0.82	-0.6	-0.77	NS	5.83 ± 0.90	-0.08	-0.50	-0.08	-0.41	NS

Liver parameters												

ALT [U/l]	25.37 ± 8.49	-1.53	+2.82	-1.65	-0.53	NS	29.17 ± 12.69	-1.79	+0.35	-4.30	-3.79	NS
AST [U/l]	25.38 ± 8.90	-1.04	-0.42	-3.15	-3.18	NS	25.33 ± 6.07	+2.69	-0.65	-2.95	-1.90	NS
Alkaline Phosphatase [U/l]	84.65 ± 22.06	-8.54	-6.76	-4.43	+0.57	*	80.33 ± 15.65	+0.34	+2.5	-2.16	+1.00	NS
GGTP [mmol/l]	20.27 ± 14.15	-5.05	-7.05	-7.16*	-5.94	*	16.83 ± 6.41	-2.66	-2.16	-2.00	-3.83	NS
Total bilirubin [*μ*mol/l]	7.64 ± 2.13	+2.03	+2.38	+2.39	+0.88	NS	9.81 ± 5.61	+1.09	+0.92	-1.03	-0.76	NS

Abbreviations: PD – Paleo diet; CD – control diet; × – arithmetic mean; SD – standard deviation; 1–2; 1–3; 1–4; 1–5 – differences between particular series; F – Friedman’s Test; BM – body mass; BMI – body mass index; FM – fat mass; FFM – fat free mass; MM – muscle mass; BMC – body mineral content; BMD – body mass density; HGB – haemoglobin; HCT – hematocrit; MCHC – mean corpuscular hemoglobin concentration; MCH – mean corpuscular haemoglobin; MCV – mean corpuscular volume; RDW-CV – red blood cell distribution with coefficient of variation; PDW – platelet distribution width; P-LCR – platelet-large cell ratio; MPV – mean platelet volume; PCT – procalcitonin; TC – total cholesterol; HDL-C – high-density lipoprotein cholesterol; LDL-C – low-density lipoprotein cholesterol; TG – triglycerides; FFA – free fatty acids; b-HB – beta-hydroxybutyrate; e-GFR – estimated glomerular filtration rate; ALT – alanine aminotransferase; AST – aspartate aminotransferase; GGTP – gamma-glutamyl transferase; italics-changes between 1–5 series;

* – test probability at the level of p < 0.05 intragroup;

** – p < 0.01;

# – test probability at the level of p < 0.05 intergroup; ## – p < 0.01 in U-Mann test.

**SUPPLEMENTARY TABLE 2:** Anaerobic and aerobic capacity. Data are reported as arithmetic mean, standard deviation and point of change (absolute changes).

	PD (n = 9)				CD (n = 6)			

	1x ± SD	1–3	1–5	F	1x ± SD	1–3	1–5	F
Wingate test on upper limbs								
Wt [kJ]	10.36 ± 1.04	-0.48	-1.01*	*	10.25 ± 1.02	+0.28	-0.01	NS
Wt [J ∙ kg^−1^]	111.85 ± 10.55	-2.07	-5.07	NS	111.33 ± 7.87	+5.67	+4.84	NS
MAP [W]	595.77 ± 71.92	-29.90	-59.10**	*	601.17 ± 67.63	+13.33	-0.017	NS
MAP [W ∙ kg^−1^]	6.41 ± 0.71	-0.12	-0.30	NS	6.52 ± 0.42	+0.29	+0.26	NS
Pmean [W]	517.97 ± 52.03	-24.41	-50.19*	**	512.33 ± 51.15	+14.17	0	NS
Pmean [W ∙ kg^−1^]	5.59 ± 0.56	-0.10	-0.26	NS	5.57 ± 0.40	+0.26	+0.23	NS
t_r_ [s]	5.26 ± 0.82	+0.20	+0.18	NS	4.86 ± 0.72	-0.13	+0.17	NS
t_m_ [s]	4.11 ± 1.43	-0.17	-0.36	NS	3.12 ± 0.74	+0.49	+0.60	NS

Wingate test on lower limbs								
Wt[kJ]	17.90 ± 1.99	-1.01	-2.15**	**	17.31 ± 2.24	-0.04	-0.88	NS
Wt [J ∙ kg^−1^]	189.91 ± 10.52	-1.69	-10.47**	**	187.33 ± 12.67	+3.63	-1.95	NS
MAP [W]	1062.35 ± 114.55	-30.68	-132.91**	**	1077.83 ± 176.07	-37.71	-38.85*	*
MAP [W ∙ kg^−1^]	11.25 ± 0.57	_+0.17	-0.65	*	11.61 ± 1.11	-0.12	+0.08	NS
Pmean [W]	895.35 ± 99.56	-50.70	-83.81**	**	865.33 ± 112.09	-2.00	-40.08	NS
Pmean [W ∙ kg^−1^]	9.49 ± 0.53	-0.09	-0.28	*	9.37 ± 0.64	+0.19	-0.02	NS
t_r_ [s]	4.04 ± 0.56	+0.01	+0.65	NS	3.72 ± 0.84	-0.21	+0.03	NS
t_m_ [s]	3.81 ± 2.21	-1.03	+0.13	NS	2.48 ± 0.52	+0.26	+0.16	NS

Aerobic capacity test								
VO_2_max [l/min]	4.31 ± 0.56	-0.01	-0.14	NS	4.22 ± 0.26	-0.01	-0.02	NS
VO_2_max [ml/kg/min]	45.70 ± 4.05	+2.3	+1.63	NS	45.83 ± 4.37	+1.48	+1.90	NS
VEmax [l/min]	149.93 ± 15.90	-5.63	-9.06	NS	154.10 ± 8.69	+2.76	-5.31	NS
TVmax [l]	2.99 ± 0.49	-0.04	-0.13*	*	2.91 ± 0.51	-0.08	-0.04	NS
RER	1.11 ± 0.04	-0.01	-0.01	NS	1.14 ± 0.04	+0.01	-0.01	NS
VE ∙ VCO_2_^−1^	30.12 ± 2.41	-0.76	-0.83	NS	30.37 ± 2.08	+0.47	-0.52	NS
HRmax [bpm]	188.59 ± 7.96	-2.14	-6.81**	**	186.00 ± 11.56	-0.23	-5.98*	*
t [min]	16.78 ± 2.02	+0.89	+0.44	NS	17.00 ± 2.39	+0.80	+0.25	NS
v [km/h]	14.70 ± 1.15	+0.43	+0.66	*	15.45 ± 1.37	+0.79	+0.52	NS

Abbreviations: PD – Paleo diet; CD – control diet; × – arithmetic mean; SD – standard deviation; 1–3; 1–5 – differences between particular series; Wt– total work performed; MAP – maximum anaerobic power; Pmean – mean anaerobic power; t_r_– the time of reaching maximum power; t_m_ – time of maintaining maximum power; VO_2_max – maximum oxygen uptake; VE max – maximum minute ventilation; TV_max_ – maximum tidal volume; RER – respiratory exchange ratio; VE ∙ VCO_2_^−1^ – respiratory equivalent carbon dioxide; HR_max_ – maximum heart rate; t-time; v – speed; F – Friedman’s Test; italics – changes between 1–5 series;

* – test probability at the level of *p <* 0.05 intragroup;

** – *p <* 0.01.
